# Large‐Scale Synthesis of Spinel Ni_x_Mn_3‐x_O_4_ Solid Solution Immobilized with Iridium Single Atoms for Efficient Alkaline Seawater Electrolysis

**DOI:** 10.1002/advs.202200529

**Published:** 2022-03-27

**Authors:** Ning Wen, Yuguo Xia, Haihua Wang, Dongpeng Zhang, Haimei Wang, Xiang Wang, Xiuling Jiao, Dairong Chen

**Affiliations:** ^1^ National Engineering Research Center for Colloidal Materials School of Chemistry and Chemical Engineering Shandong University Jinan Shandong 250100 P. R. China; ^2^ MOE Key Laboratory of Pollution Processes and Environmental Criteria/Tianjin Key Laboratory of Environmental Remediation and Pollution Control College of Environmental Science and Engineering Nankai University Tianjin 300350 P. R. China

**Keywords:** Ir single atoms, Ni_x_Mn_3‐x_O_4_, OER, seawater electrocatalyst, sol‐gel method

## Abstract

Seawater electrolysis not only affords a promising approach to produce clean hydrogen fuel but also alleviates the bottleneck of freshwater feeds. Here, a novel strategy for large‐scale preparing spinel Ni_x_Mn_3‐x_O_4_ solid solution immobilized with iridium single‐atoms (Ir‐SAs) is developed by the sol–gel method. Benefitting from the surface‐exposed Ir‐SAs, Ir_1_/Ni_1.6_Mn_1.4_O_4_ reveals boosted oxygen evolution reaction (OER) performance, achieving overpotentials of 330 and 350 mV at current densities of 100 and 200 mA cm^–2^ in alkaline seawater. Moreover, only a cell voltage of 1.50 V is required to reach 500 mA cm^–2^ with assembled Ir_1_/Ni_1.6_Mn_1.4_O_4_‖Pt/C electrode pair under the industrial operating condition. The experimental characterizations and theoretical calculations highlight the effect of Ir‐SAs on improving the intrinsic OER activity and facilitating surface charge transfer kinetics, and evidence the energetically stabilized *OOH and the destabilized chloride ion adsorption in Ir_1_/Ni_1.6_Mn_1.4_O_4_. This work demonstrates an effective method to produce efficient alkaline seawater electrocatalyst massively.

## Graphical abstract

Exploring rational strategy for large‐scale synthesis of OER electrocatalysts with high activity and structural robustness is essential to achieve industrial seawater electrocatalysts. In this work, mass‐productive Ni_x_Mn_3‐x_O_4_ solid solution immobilized with iridium single‐atoms reveals excellent OER activity and anti‐chloride corrosion characteristics, rendering it a promising seawater electrocatalyst.

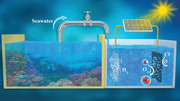

## 1 Introduction

Employing renewable electricity combined with water electrolyzers to produce hydrogen presents an appealing and sustainable strategy to combat climate changes and secure energy.^[^ ^1^, ^2^ ^]^ Principally, the overall efficiency of water electrolysis is hampered by oxygen evolution reaction (OER) in consideration of its sluggish, multistep proton‐coupled electron transfer process.^[^ ^3^ ^]^ Over the past few years, numerous works have been devoted to explore efficient OER catalysts, including transition metal oxides,^[^ ^4^, ^5^, ^6^ ^]^ (oxy)hydroxides,^[^ ^7^, ^8^, ^9^ ^]^ sulfides,^[^ ^10^, ^11^, ^12^ ^]^ phosphides,^[^ ^13^, ^14^, ^15^ ^]^ and nitrides.^[^ ^16^, ^17^, ^18^ ^]^ Moreover, part of them even prevails over the benchmark IrO_2_/RuO_2_ in the OER activity and stability,^[^ ^19^, ^20^, ^21^ ^]^ which certainly invigorates the blossom of the water electrolyzer technique. However, the freshwater feeds may become a bottleneck for large‐scale water electrolysis. Given the abundant natural resources as well as the improved ionic conductivity due to dissolved salts, seawater thereby becomes the optimal choice to alleviate this issue.

The main challenge for seawater electrolysis is the competitive active chlorine species formation reactions (ACSFRs), including the chlorine evolution reaction in low pH and chlorine oxidation reactions in high pH to generate hypochlorite.^[^ ^22^ ^]^ Thus, OER selectivity for seawater electrolysis is highly essential. According to the Pourbaix diagram for the oxygen evolution reaction and chloride chemistry, OER selectivity over ACSFRs can be achieved with the maximum potential difference of 490 mV in the high pH value.^[^ ^23^, ^24^ ^]^ Therefore, considering the kinetics and standard potentials, an alkaline environment is more favorable to avoid hypochlorite formation during seawater electrolysis. Besides, considering the industrial requirement for delivering large current density (>500 mA cm^–2^) under seawater electrolyzers, the design of high‐performance seawater electrocatalysts is still challenging.^[^ ^25^ ^]^

The primary principle to design seawater electrocatalysts is to improve the corrosion resistance of electrocatalysts/seawater interfaces, that is, to stabilize –OOH formation or destabilize chloride ion adsorption, thereby enhancing the OER selectivity.^[^ ^26^ ^]^ Unfortunately, the recently reported strategies for seawater electrocatalysts design are still involved in doping modification, defects construction, and surface engineering to lower the *d*‐band centers, scilicet mainly concerning the improvement of the OER activity.^[^ ^27^, ^28^, ^29^, ^30^ ^]^ The up‐to‐date seawater electrocatalysts also basically follow those in water electrolyzers, whereas few can meet the industrially mandated overpotential of 300 mV at 500 mA cm^–2^ with a cell voltage of below 1.60 V. Besides, apart from the prerequisite OER efficiency and stability, industrial seawater electrolyzer also require the electrocatalysts that can be easily scaled up, which is hardly achieved by template‐based synthesis or exfoliation process.^[^ ^31^, ^32^ ^]^ Therefore, developing innovative seawater electrocatalysts with efficient OER activity and high selectivity, and mass‐productive characteristics is of great necessity.

Herein, inspired by the impressive selectivity and stability of Mn‐based oxides in the acidic electrolyte,^[^ ^33^, ^34^ ^]^ we consider that the Mn‐based oxides may also represent good selectivity in the alkaline electrolyte due to the similar chlorine ions adsorbing process by surface polarization. Generally, the intrinsic activity of OER electrocatalysts can be tuned by elemental doping or introducing vacancies.^[^ ^35^, ^36^ ^]^ Herein, benefitting from the structural similarity of cubic spinel NiMn_2_O_4_ and Ni_2_MnO_4_, we consider that tuning the OER activity of NiMn‐based oxide can be possibly achieved by preparing Ni_x_Mn_3‐x_O_4_ solid solution. Furthermore, massively producing Ni_x_Mn_3‐x_O_4_ through the sol‐gel process is experimentally feasible.^[^ ^37^ ^]^ Therefore, we employed cubic spinel Ni_x_Mn_3‐x_O_4_ solid solution in the seawater electrolyzer, which manifested excellent chlorine oxidation resistance. Meanwhile, we introduced Ir single atoms into Ni_x_Mn_3‐x_O_4_ to further enhance intrinsic OER performance and increase the number of active sites to alleviate the effect of possible insoluble precipitates. The Ir_1_/Ni_1.6_Mn_1.4_O_4_ reveals low overpotentials of 330 and 350 mV to achieve the current densities of 100 and 200 mA cm^–2^. Moreover, only a cell voltage of 1.50 V is required to reach 500 mA cm^–2^ with assembled Ir_1_/Ni_1.6_Mn_1.4_O_4_‖Pt/C electrode pair under the industrial operating condition, demonstrative of the feasibility for Ir_1_/Ni_1.6_Mn_1.4_O_4_ employed in alkaline seawater electrolyzer. In addition, the surface structural regulation and the plausible mechanism of Ir single atoms on the enhanced OER performance are also discussed.

## 2 Results and Discussion

### 2.1 Synthesis and Morphological Characterizations of Ir_1_/Ni_1.6_Mn_1.4_O_4_ Nanocrystals

**Figure 1:**
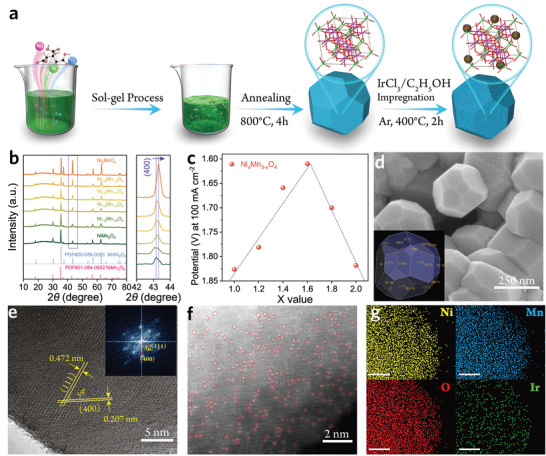
a) The schematic diagram for the synthetic procedure of the Ir_1_/Ni_1.6_Mn_1.4_O_4_. b) The XRD patterns of Ni_x_Mn_3‐x_O_4_ (*x* = 1–2). The inset corresponds to a local magnified peak assigned to the (400) plane. c) The volcano plot of voltages obtained at 100 mA cm^–2^ for Ni_x_Mn_3‐x_O_4_. d) SEM image of Ni_1.6_Mn_1.4_O_4_. The inset is the reconstructed shape based on the BFDH theory. e) HR‐TEM image of Ni_1.6_Mn_1.4_O_4_. The inset reveals the corresponding FFT image. f) HAADF‐STEM image of 0.459%Ir‐Ni_1.6_Mn_1.4_O_4_, indicative of the Ir‐SAs. g) The HAADF‐STEM energy dispersive X‐ray mapping. The bars represent 10 nm.

**Figure** **1**a displays the typical procedures to prepare Ni_1.6_Mn_1.4_O_4_ nanocrystals immobilized with Ir single atoms, which involves three steps, i.e., sol‐gel process, annealing treatment, and impregnation. Given the structural similarity and ingredients adjustable characteristic in NiMn_2_O_4_ and MnNi_2_O_4_ (Figure S1, Supporting Information), spinel Ni_x_Mn_3‐x_O_4_ solid solutions with variable *x* (*x* = 1–2) are prepared. The XRD patterns confirm the successive structural changes in Ni_x_Mn_3‐x_O_4_ with the proportional increase of the Ni component, indicating the successful preparation of ingredients adjustable spinel Ni_x_Mn_3‐x_O_4_ solid solution (Figure 1b). Given the smaller atomic radius of Ni relative to Mn, the lattice parameter should be diminished with the increase of Ni ratio in Ni_x_Mn_3‐x_O_4_ solid solutions, and the Bragg diffraction position for the same crystal plane should shift to a higher grazing angle with the increase of Ni ratio as confirmed by the enlarged XRD pattern. The polarization curves of Ni_x_Mn_3‐x_O_4_ solid solutions with variable *x* are first measured (Figure 1c; Figure S2, Supporting Informaton), revealing a volcanic trend, and Ni_1.6_Mn_1.4_O_4_ locates at the crest of the volcano plot. Besides, the surface charge analysis for Ni_x_Mn_3‐x_O_4_ reveals a similar tendency as in the polarization curves, indicative of the effect of Ni and Mn contents in tuning the OER performance mainly embodied in surface charge (Figure S3, Supporting Information). Therefore, we consider the surface electronic structure of Ni_1.6_Mn_1.4_O_4_ is most beneficial to the water oxidation process. The morphologies of Ni_x_Mn_3‐x_O_4_ are investigated by scanning electron microscopy (SEM)(Figure S4, Supporting Information), where no distinct morphological changes for Ni_x_Mn_3‐x_O_4_ nanoparticles are observed, which indicates that the morphology of Ni_x_Mn_3‐x_O_4_ nanocrystal is not dependent on the Ni/Mn ratio in Ni_x_Mn_3‐x_O_4_. The typical SEM image of Ni_1.6_Mn_1.4_O_4_ has revealed in Figure 1d, and the nanocrystals display an octadecahedron configuration. Given the similarity of lattice parameters in the Ni_1.6_Mn_1.4_O_4_ model (Figure S5, Supporting Information), the octadechedral nanocrystals mainly consist of {011} families of lattice planes as well as a small proportion of {100} as confirmed by the reconstructed shape. Meanwhile, the theoretical model to simulate Ni_1.6_Mn_1.4_O_4_ is also verified by Rietveld analysis (Figure S6, Supporting Information), which fits well with the experimental XRD patterns, illustrative of the rationality of the model. The high‐resolution transmission electron microscopy (HR‐TEM) image further confirms the dominant ($0\bar{1}1$) crystal plane (Figure 1e). The loading amounts of Ir atoms are primarily optimized (Figure S7, Supporting Information), and Ir with a mass fraction of 0.459% in Ir/Ni_1.6_Mn_1.4_O_4_ reveals the best OER performance (The Ir atom mass fraction in Ir‐Ni_1.6_Mn_1.4_O_4_ is determined by Inductively coupled plasma mass spectrometry, Table S1, Supporting Information). Besides, to elucidate the synergistic effect between Ni_x_Mn_3‐x_O_4_ and Ir atoms, the polarization curves of Ir/Ni_x_Mn_3‐x_O_4_ and Ir cluster/Ni_1.6_Mn_1.4_O_4_ are measured (Figure S8, Supporting Information), which both confirms the superior OER performance of 0.459%Ir‐Ni_1.6_Mn_1.4_O_4_ and highlights the importance of Ni_1.6_Mn_1.4_O_4_ substrates and proper Ir addition. Moreover, the distribution of Ir atoms in 0.459%Ir/Ni_1.6_Mn_1.4_O_4_ is further explored by the high‐angle annular dark‐field scanning transmission electron microscopy (HAADF‐STEM) image (Figure 1f), where the Ir atoms are discretely anchored on the surface of Ni_1.6_Mn_1.4_O_4_ nanocrystals. The STEM‐energy dispersive X‐ray mapping also evidences the atomically dispersed Ir atoms in 0.459%Ir/Ni_1.6_Mn_1.4_O_4_ (hereafter donated as Ir_1_/Ni_1.6_Mn_1.4_O_4_) (Figure 1g), suggesting the characteristics of the single‐atom catalyst.

**Figure 2:**
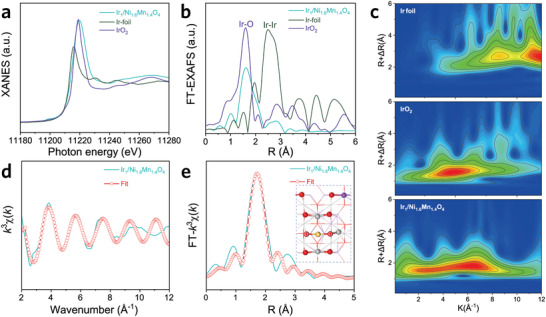
a) Ir *L*3‐edge XANES spectra of Ir_1_/Ni_1.6_Mn_1.4_O_4_, standard Ir foil, and commercial IrO_2_.b) The magnitude of the Fourier transforms of *k* ^3^‐weighted Ir *L*3‐edge EXAFS functions in Ir_1_/Ni_1.6_Mn_1.4_O_4_, Ir foil, and IrO_2_. c) The corresponding wavelet transforms for the *k* ^3^‐weighted Ir *L*3‐edge EXAFS signals of Ir_1_/Ni_1.6_Mn_1.4_O_4_ compared to Ir foil and IrO_2_. d,e) Ir *L*3‐edge extended EXAFS and fitting results of Ir_1_/Ni_1.6_Mn_1.4_O_4_ in *K* (g) and *R*(h) spaces. Inset corresponds to the schematic model of Ir_1_/Ni_1.6_Mn_1.4_O_4_.

The surface elemental charge state changes involved in Ir_1_/Ni_1.6_Mn_1.4_O_4_ were first measured by X‐ray photoelectron spectroscopy (Figure S9, Supporting Information). Generally, the position of binding energy for a certain atom is affected by its coordination environment or valence state.^[^ ^38^ ^]^ Herein, the Ir 4*f* peak in Ir_1_/Ni_1.6_Mn_1.4_O_4_ shifts toward lower binding energy relative to IrO_2_, indicative of its lower valence state than that in IrO_2_.^[^ ^39^ ^]^ We further employed the X‐ray absorption near‐edge structure (XANES) spectra and density functional theory (DFT) calculations to illustrate the coordination information of Ir atoms in the Ir_1_‐Ni_1.6_Mn_1.4_O_4_. As displayed in **Figure** **2**a, the absorption edge position for Ir_1_/Ni_1.6_Mn_1.4_O_4_ locates between Ir foil and IrO_2_, illustrative of the valence state of the Ir atom in Ir_1_/Ni_1.6_Mn_1.4_O_4_ situating between 0 and +4. Simultaneously, the possible absorption geometries for Ir binding on Ni_1.6_Mn_1.4_O_4_ are theoretically calculated (Figure S10, Supporting Information), and the Ir atom coordinated with two adjacent O displays the most thermodynamical configuration. The electronic redistribution in Ir_1_/Ni_1.6_Mn_1.4_O_4_ is analyzed by Bader charge, where Ir represents +3.7125|e| and in accordance with the XANES result. Besides, the coordination environment around Ir in Ir_1_/Ni_1.6_Mn_1.4_O_4_ was further identified by extended X‐ray absorption fine structure (EXAFS) spectra and wavelet transform analysis. The Fourier transformed *k* ^3^‐weighted EXAFS curves reveal the prominent peak around 1.6 Å (Figure 2b), which can be ascribed to the Ir‐O bond, and no Ir–Ir bond at 2.5 Å is observed, indicative of the atomically dispersed Ir sites in Ir_1_/Ni_1.6_Mn_1.4_O_4_.^[^ ^40^ ^]^ Meanwhile, only one intensity maximum at 6 Å^–1^ can be observed from the wavelet transformed contour plots for Ir_1_/Ni_1.6_Mn_1.4_O_4_ (Figure 2c), which is assigned to Ir–O coordination without Ir–Ir signal. The FT‐EXAFS results referenced to our theoretical most stable Ir_1_/Ni_1.6_Mn_1.4_O_4_ geometry are further fitted in *k* and *R* spaces to investigate the coordination configuration. As revealed in Figure 2d,e, the FT‐EXAFS fitting results comply well with the measured results (Table S2, Supporting Information), and the coordination number for Ir‐O is 1.9, which evidences the Ir‐O_2_ center in Ir_1_/Ni_1.6_Mn_1.4_O_4_, also consistent with our DFT calculated result.

### 2.2 Electrocatalytic OER Performance of Ir_1_/Ni_1.6_Mn_1.4_O_4_

**Figure 3:**
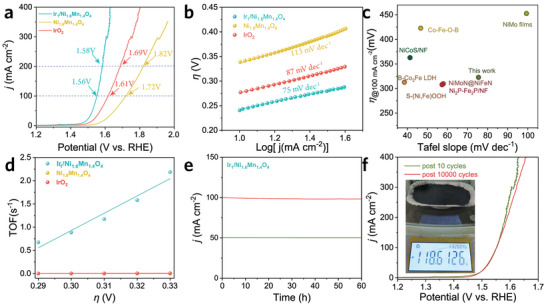
a) OER polarization curves with *iR* correction. b) Tafel slope. c) Comparison of Tafel slopes and overpotentials required to achieve the current density of 100 mA cm^–2^.^[^ ^28^, ^43^, ^44^, ^45^, ^46^, ^47^, ^48^ ^]^ d) TOF values. e) The chronoamperometry curves. f) OER polarization curves measured during the ADT test. Electrolyte: 0.5 m KOH+ natural seawater; scan rate: 10 mV s^–1^; temperature: 25 °C. Inset corresponds to the optical image of Ir_1_/Ni_1.6_Mn_1.4_O_4_ in a typical synthetic procedure.

The electrocatalytic performances of Ir_1_/Ni_1.6_Mn_1.4_O_4_ for OER are investigated in a standard three‐electrode configuration in alkaline seawater. The salinity and OER polarization curves for Ir_1_/Ni_1.6_Mn_1.4_O_4_ in different alkaline seawater are supplemented in Figure S11 (Supporting Information), which illustrates that 0.5 m KOH + seawater exhibits comparable OER performance to 1 m KOH and is thereby employed as the electrolyte. **Figure** **3**a displays the linear sweep voltammetry (LSV) polarization curves of pristine Ni_1.6_Mn_1.4_O_4_ and Ir_1_/Ni_1.6_Mn_1.4_O_4_, and Ir_1_/Ni_1.6_Mn_1.4_O_4_ exhibits a lower onset potential (Figure S12, Supporting Information) and only requires an *i*R‐corrected 1.56 V_RHE_ and 1.58 V_RHE_ (*η* of 330 and 350 mV, respectively) to achieve a current density of 100 and 200 mA cm^–2^, which is superior to that of pristine Ni_1.6_Mn_1.4_O_4_ (*η* of 490 and 590 mV, respectively) and commercial IrO_2_ (*η* of 380 and 460 mV), as well as below the 490 mV overpotential required to trigger chloride oxidation to hypochlorite. Meanwhile, the LSV polarization curves without *i*R compensation are supplemented in Figure S13 (Supporting Information), which reveals a similar tendency and illustrates the good OER performance of Ir_1_/Ni_1.6_Mn_1.4_O_4_. In addition, the Tafel slope of Ir_1_/Ni_1.6_Mn_1.4_O_4_ is decreased from 113 to 75 mV dec^–1^, suggesting improved reaction kinetics (Figure 3b). As shown in Figure 3c, the Ir_1_/Ni_1.6_Mn_1.4_O_4_ represents comparable electrocatalytic OER performance in alkaline seawater relative to the other benchmarking catalysts. The turnover frequency (TOF) and mass activity are further calculated. The TOF value of Ir_1_/Ni_1.6_Mn_1.4_O_4_ exceeds Ni_1.6_Mn_1.4_O_4_ and IrO_2_ over 50 to 82‐folds, respectively (Figure 3d). Meanwhile, when normalized to active mass, the activity of Ir_1_/Ni_1.6_Mn_1.4_O_4_ still reveals more than four times enhancement relative to Ni_1.6_Mn_1.4_O_4_ (Figure S14, Supporting Information), highlighting the enhanced intrinsic electrocatalytic activity due to the Ir single atoms. The electrocatalytic robustness of Ir_1_/Ni_1.6_Mn_1.4_O_4_ in alkaline seawater is evaluated by accelerated degradation test (ADT) and long‐term chronoamperometry. The OER activities remain nearly unchanged with continuous oxygen release for 60 h at current densities of 50 and 100 mA cm^–2^ (Figure 3e), and no evident changes are observed in the LSV polarization curve even after 10 000 cycles (Figure 3f), both indicative of the excellent durability of Ir_1_/Ni_1.6_Mn_1.4_O_4_. Moreover, no noticeable phase structural and morphological changes of Ir_1_/Ni_1.6_Mn_1.4_O_4_ after long‐term *I–t* test (Figure S15, Supporting Information), further evidencing its good structural stability in alkaline seawater. Particularly, to investigate the structural stability of Ir single atom on Ni_1.6_Mn_1.4_O_4_, Raman spectra were measured (Figure S16, Supporting Information), where the peak position assigned to Ir‐O vibration is nearly unchanged, illustrative of its coordination structural robustness.^[^ ^41^ ^]^ Besides, the stability of the Ir single atom in Ir_1_/Ni_1.6_Mn_1.4_O_4_ is also theoretically calculated, where its binding energy (−7.36 eV, Figure S10, Supporting Information) is much smaller than the corresponding cohesive energy (6.94 eV atom^−1^),^[^ ^42^ ^]^ indicative of the thermodynamical stability of Ir single atoms. Notably, benefitting from the sol‐gel process for preparing Ni_1.6_Mn_1.4_O_4_, Ir_1_/Ni_1.6_Mn_1.4_O_4_ can be easily mass‐produced in the hectogram scale (inset of Figure 3f), rendering the industrial application possible. Meanwhile, the gas evolution during the OER is measured by gas chromatography to subsequently calculate the Faradaic efficiency (FE) (Figure S17, Supporting Information). The ca. 100% efficiency for Ir_1_/Ni_1.6_Mn_1.4_O_4_ indicates the polarization current solely consumed in the OER process.

### 2.3 Electrocatalytic Performance for Overall Water Splitting

**Figure 4:**
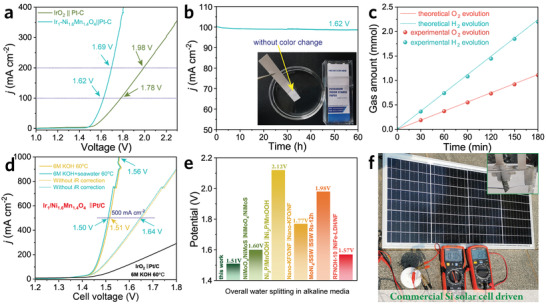
a) LSV curves of the Ir_1_/Ni_1.6_Mn_1.4_O_4_‖Pt/C electrode pairs for overall water splitting. b) Current density versus time curves at an applied voltage of 1.62 V. The inset is the potassium iodide starch paper immersed in the electrolyte after 60 h *J–t* test. c) Experimental and theoretical gas evolution of Ir_1_/Ni_1.6_Mn_1.4_O_4_‖Pt/C electrode pairs versus time. Electrolyte: natural seawater + 0.5 m KOH; scan rate: 10 mV s^–1^; temperature: 25 °C. d) LSV curves of the Ir_1_/Ni_1.6_Mn_1.4_O_4_‖Pt/C electrode pairs in 6 m KOH + seawater at 60 °C. e) Comparison of operation voltages required to achieve the current density of 500 mA cm^–2^ with benchmarking works.^[^ ^50^, ^51^, ^52^, ^53^, ^54^ ^]^ f) Optical image of Ir_1_/Ni_1.6_Mn_1.4_O_4_‖Pt/C electrode pairs driven by silicon PV in 0.5 m KOH + seawater.

The Ir_1_/Ni_1.6_Mn_1.4_O_4_ assembled with commercial Pt/C electrode is constructed as a two‐electrode system for overall water splitting in alkaline seawater. As revealed in **Figure** **4**a, the Ir_1_/Ni_1.6_Mn_1.4_O_4_‖Pt/C catalytic couple affords current densities of 100 and 200 mA cm^–2^ at 1.62 and 1.69 V (*η* of 390 and 460 mV, respectively). In contrast, the commercial IrO_2_‖Pt/C electrode pairs achieve the same current densities at 1.78 V and 1.98 V, respectively. The electrochemical stability for overall water splitting is investigated by long‐term *J–t* measurement (Figure 4b). No obvious decay is observed after continuous operation for 60 h, illustrative of the robust structural stability. In addition, the post‐OER electrolyte is monitored by potassium iodide starch paper, where no color change appears, suggesting the excellent OER selectivity and anti‐corrosion property in alkaline seawater circumstances. The gas evolutions in the two‐electrode cell were also measured by gas chromatography (Figure 4c). Especially, the oxygen release rate approaches its theoretical value, which illustrates the high electron utilization efficiency with FE of O_2_ above 99.0% (Figure S18, Supporting Information). In addition, the industrial application requires a large current density (e.g., 500 and 1000 mA cm^–2^) in concentrated alkaline circumstances (typically 6 m KOH solution at 60 ˚C).^[^ ^49^ ^]^ The Ir_1_/Ni_1.6_Mn_1.4_O_4_‖Pt/C catalytic couple affords current densities of 500 and1000 mA cm^–2^ at only1.51 and 1.56 V in 6 m KOH electrolyte (Figure 4d), respectively, superior to the IrO_2_‖Pt/C couple (The HER performance of Pt–C electrode is also supplemented in Figure S19, Supporting Information). A similar polarization curve is achieved for the Ir_1_/Ni_1.6_Mn_1.4_O_4_‖Pt/C catalytic couple in alkaline seawater (*η*_500_ = 1.50 V, *η*_1000_ = 1.56 V), indicating the feasibility of employing alkaline seawater for electrocatalytic water splitting. Given the operational stability as importing metric, the Ir_1_/Ni_1.6_Mn_1.4_O_4_‖Pt/C catalytic couple can maintain the excellent electrocatalytic activity at 1.50 V with a large current density of 500 mA cm^–2^ over 50 h without apparent degradation in 6 m KOH + seawater at 60 ˚C (Figure S20, Supporting Information). In addition, compared to other benchmarking electrocatalysts with large current densities (Figure 4e), the operation voltage for the Ir_1_/Ni_1.6_Mn_1.4_O_4_‖Pt/C catalytic couple is still dominant, which renders it a promising industrial candidate for overall water splitting. Besides, the ideal power supply in the coastal areas could be abundant solar energy.^[^ ^22^ ^]^ Therefore, the PV‐electrolysis system comprising a commercial Si PV module connected to the two‐electrode cell is constructed. As revealed in Figure 4f, the seawater electrolyzer driven by a commercial Si solar cell achieves an impressively high current of 1.04 A under a photovoltage of 2.85 V without generating hypochlorite, indicating the feasibility for electrocatalytic water splitting with the PV‐electrolysis system.

### 2.4 Origin of the Enhanced OER Mechanism

**Figure 5:**
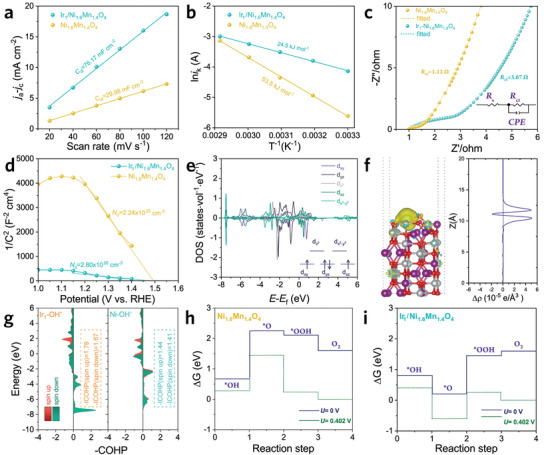
a) The current density difference (*j*_a_−*j*_c_) that plotted against scan rates. b) Arrhenius plot of the kinetic current at *η* = 200 mV. c) Nyquist plot measured with an open‐circuit voltage of 1.20 V and corresponding equivalent circuit in alkaline seawater (0.5 m KOH + seawater). d) Mott–Schottky plots. e) Calculated projected density of states of Ir atom. f) Charge density difference and the plane‐average electron difference. g) Projected crystal orbital Hamilton populations (COHP) of OH^–^ absorbing on Ni_1.6_Mn_1.4_O_4_ and Ir_1_/Ni_1.6_Mn_1.4_O_4_. The free energy diagram for the intermediates' evolution on h) Ni_1.6_Mn_1.4_O_4_($0\bar{1}1$) and i) Ir_1_/Ni_1.6_Mn_1.4_O_4_.

To explore the origin of the efficient OER activity of the Ir_1_/Ni_1.6_Mn_1.4_O_4_ catalyst, we further investigate its pseudocapacitance and charge transfer characteristics. Given the high dependence of electrochemical activity on the number of active sites, the electrochemical surface areas (ECSA) estimated by the electrochemical double‐layer capacitances (*C*_dl_) are calculated (**Figure** **5**a), where the *C*_dl_ value of Ir_1_/Ni_1.6_Mn_1.4_O_4_ (76.17 mF cm^–2^), much higher than that of Ni_1.6_Mn_1.4_O_4_ (29.98 mF cm^–2^), suggesting that the enhanced OER activity is contributed mainly by the increased number of active sites. To further determine whether the enhanced OER activity is solely contingent on the increased active sites, the ECSA‐normalized OER polarization curves are calculated (Figure S21, Supporting Information). The Ir_1_/Ni_1.6_Mn_1.4_O_4_ still possesses lower overpotentials, illustrative of the Ir single atoms not only creating new active sites but also enhancing its intrinsic activity. To access the activation energy (*E*_a_) of the surface oxygen evolution reaction, which can be extracted from the slope of the Arrhenius plot, we measured the variation of current density along with the temperature (Figure 5b).^[^ ^55^ ^]^ The Ir_1_/Ni_1.6_Mn_1.4_O_4_ reveals a much smaller *E*_a_ value (24.5 kJ mol^–1^) compared to that of Ni_1.6_Mn_1.4_O_4_(53.5 kJ mol^–1^), which demonstrates the importance of Ir single atoms in accelerating the surface OER kinetics. Besides, the charge transport behavior of Ir_1_/Ni_1.6_Mn_1.4_O_4_ is investigated by electrochemical impedance spectra (EIS) measurements. As depicted in Figure 5c, the decreased interfacial charge transfer resistance (*R*_ct_) in Ir_1_/Ni_1.6_Mn_1.4_O_4_ illustrates its increased charge transfer rate. Moreover, the charge‐carrier density deduced from the Mott–Schottky plot is calculated to evaluate the charge‐carrier concentration (Figure 5d). The *N*_d_ value of Ir_1_/Ni_1.6_Mn_1.4_O_4_ (2.80 × 10^26 ^cm^–3^) is about tenfold that of pristine Ni_1.6_Mn_1.4_O_4_(2.24 × 10^25 ^cm^–3^), indicative of the crucial role of Ir single atoms in charge transfer.

To elucidate the underlying reasons for the enhanced OER activity in Ir_1_/Ni_1.6_Mn_1.4_O_4_, we further investigate the variations of charge distribution and the OER evolution route with Ir single atoms modification. The electronic structure of Ni_1.6_Mn_1.4_O_4_ modulated by the Ir atom is primarily investigated. Both the *d*_xy_ and *d*_xz_ states of Ir are half occupied (Figure 5e), demonstrating the high‐spin state of the Ir atom in Ir_1_/Ni_1.6_Mn_1.4_O_4_ and indicating its improved conductivity.^[^ ^56^ ^]^ Besides, as revealed in the optimized coordination model (Figure 5f), charge redistribution appears on the surface of Ir_1_/Ni_1.6_Mn_1.4_O_4_, where the Ir atom loses few electrons, and the delocalized electron mainly accumulates around Ir and adjacent O atoms. We also calculated the crystal orbital Hamilton populations (COHP) of OH^–^ absorbing on Ni_1.6_Mn_1.4_O_4_ and Ir_1_/Ni_1.6_Mn_1.4_O_4_ (Figure 5g). The integral crystal orbital Hamilton populations (ICOHP) results revealed that the intensity of OH^–^ bonding on the Ir_1_ site (‐ICOHP = 1.79/1.67) is stronger than that on Ni_1.6_Mn_1.4_O_4_ (‐ICOHP = 1.44/1.41), indicating that the easier absorption of OH^–^ to generate *OH in the OER process on Ir_1_/Ni_1.6_Mn_1.4_O_4_.^[^ ^57^ ^]^ Additionally, we calculated the Gibbs free energy evolution of the crucial intermediates to illustrate the potential‐determining step in the water oxidation process (Figures S22 and S23, and Tables S2–S5, Supporting Information). Notably, except for the reduced largest Gibbs free energy difference in Ir_1_/Ni_1.6_Mn_1.4_O_4_ (Δ*G* = 0.404 eV) compared to that of Ni_1.6_Mn_1.4_O_4_ (Δ*G* = 1.449 eV), the rate‐determining step (RDS) has changed from the *O formation process to the *OOH formation process (Figure 5h,i), which is considered to be the fundamental reason for the reduced overpotential and improved OER kinetics in Ir_1_/Ni_1.6_Mn_1.4_O_4_. Meanwhile, the aggressive chloride ions in seawater corrode electrocatalysts generally through metal chloride‐hydroxide formation mechanisms, which involve three steps, that is, chloride ion adsorption, dissolution by further coordination, and conversion from chloride to hydroxide. Herein, we first calculate the adsorption energies for chlorine ions on Ni_1.6_Mn_1.4_O_4_ and Ir_1_/Ni_1.6_Mn_1.4_O_4_, which is determined to be 3.159 and 4.355 eV (Figure S24 and Table S6, Supporting Information), respectively. The positive adsorption energies indicate the thermodynamical unfavorable for chlorine absorbing on Ni_1.6_Mn_1.4_O_4_ and Ir_1_/Ni_1.6_Mn_1.4_O_4_, which accounts for the excellent OER selectivity over hypochlorite generation in alkaline seawater.

## 3 Conclusions

In summary, we developed a simple and effective approach to mass‐productively synthesize Ir‐SAs immobilized Ni_1.6_Mn_1.4_O_4_ for alkaline seawater electrolysis. The effects of Ir‐SAs are thoroughly investigated by experimental characterizations and theoretical calculations. The EXAFS results and binding energy calculations confirm the Ir‐O_2_ configuration. The surface electronic structural analysis and Gibbs free energy evolution for OER intermediates demonstrate the crucial factor of Ir‐SAs in enhancing intrinsic OER activity and improving the interfacial charge transfer. Benefitting from the Ir‐SAs as well as the specific Ni_1.6_Mn_1.4_O_4_($0\bar{1}1$) surface on stabilizing *OOH intermediate and destabilizing Cl^–^ absorption, the Ir_1_/Ni_1.6_Mn_1.4_O_4_ exhibits impressive alkaline seawater OER activity, achieving overpotentials of 330 and 350 mV at current densities of 100 and 200 mA cm^–2^. By evaluating its overall water‐splitting performance, a cell voltage of only 1.50 V is required to reach 500 mA cm^–2^ with assembled Ir_1_/Ni_1.6_Mn_1.4_O_4_‖Pt/C electrode pair under the industrial operating condition, superior to up‐to‐date alkaline seawater electrolyzer. The sol‐gel strategy in this manuscript presents a promising way to massively prepare highly efficient electrolytes in alkaline seawater, which may inspire more excellent works on developing highly efficient alkaline seawater electrolyzers.

## 4 Experimental Section

### Synthesis of Ni_x_Mn_3‐x_O_4_ Solid Solution

The NiMn_2_O_4_ powders were synthesized by a sol–gel method. Typically, 5 mmol of Ni(NO_3_)_2_·6H_2_O, 10 mmol of Mn(NO_3_)_2_·4H_2_O, and 50 mmol of citric acid were dissolved into 20 mL of distilled water. Subsequently, the NH_3_·H_2_O was added dropwise into the above solution that was placed in an ice‐water bath until the pH reached 8. Then, the above mixture was thermally treated at 60 °C for 12 h to get a dry gel. Finally, the dry gel was transferred to the muffle furnace and heated at 800 °C for 4 h with a heating rate of 5 °C·min^–1^.^[^ ^58^ ^]^ The Ni_x_Mn_3‐x_O_4_ solid solution samples were prepared by altering the ratio of the precursors.

### Synthesis of Ir_1_/Ni_1.6_Mn_1.4_O_4_ Nanocrystals

Typically, 0.5 g of Ni_1.6_Mn_1.4_O_4_ was added into 10 mL ethanol solution with ultrasonic treatment for 5 min to obtain a well‐dispersed mixture. Then, 114.6 µL IrCl_3_ solution (50 mg mL^–1^) was added into the above dispersion dropwise under magnetic stirring and heated at 50 °C to completely volatilize the ethanol. The obtained black powder was then transferred into a ceramic boat and thermal‐treated at 400 °C for 2 h under an Ar atmosphere. After cooling down to room temperature naturally, the powders were collected and denoted as Ir_1_/Ni_1.6_Mn_1.4_O_4_.

### Preparation of Ir_1_/Ni_1.6_Mn_1.4_O_4_ and Pt–C Electrodes

Typically, 80 mg of catalyst and 10 mg of acetylene black were placed in a mortar. Subsequently, 50 µL of 5 wt.% Nafion and 25 µL of N‐methylpyrrolidone were added dropwise. Then, a uniform ink was obtained after grinding for 30 min. The above suspension was spread on the Ti net and dried at 60 °C. The catalyst loading was ≈4mg cm^–2^_._

### Characterization

The crystalline structures were analyzed by powder X‐ray diffraction (D8, Bruker AXS) with Cu K*α* radiation (*λ* = 1.5418 Å). The morphology and microstructure were characterized using SEM (Hitachi, SU8010), high‐resolution TEM (JEM‐1011, JEOL), and spherical aberration‐corrected TEM (JEM‐ARM200F). The XPS (EscaLab 250Xi, Thermo scientific) technique with 30.0 eV pass energy and an Al K*α* line excitation source was employed to identify the elemental compositions and bonding information. The gas evolutions were analyzed by gas chromatography (3420A, Beifen‐Ruili Co. Ltd., China).

### Electrochemical Measurements

The measurements were conducted on a CHI760E electrochemical workstation with a standard three‐electrode system. The as‐prepared samples were employed as working electrodes with an average catalyst loading of ≈4 mg cm^–2^, and graphite rod and Hg/HgO electrode (1.0 m KOH) were used as counter and reference electrodes, respectively. All the measurements were carried out in O_2_‐saturated electrolyte. The measured potentials were calibrated to the reversible hydrogen electrode (RHE) by the equation: *E*_RHE_ = *E*_Hg/HgO _+ 0.098 + 0.059 × pH. The EIS spectra were recorded in 0.5 m KOH+seawater at open‐circuit potential with the frequency range from 1 MHz to 0.1 Hz. Mott−Schottky plots were recorded from 0 to 1.6 V versus Hg/HgO reference electrode with the frequency of 1 kHz.

### Methods for Faradaic Efficiency and TOF Calculation

Details about the calculations of Faradic efficiency and turnover frequency (TOF) are shown below:

(1) η=(m×n×F)/(I×t)

where *η is* the Faradic efficiency, *m* is the actual molar number of H_2_ or O_2_, *n* is the number of reactive electrons, *F* is Faraday's constant (96 485.3 C mol^–1^), *I* is the current, and *t* is time.

(2) TOF=(j×S)/(N×F×n)

where *j* represents the measured current density, *S* represents the surface area of the electrode (typically 1 cm^2^), *N* represents the number of electrons required per mole of gas (H_2_ or O_2_), *F* is the Faraday's constant F (96 485.3 C mol^–1^), and *n* is the moles of metal atoms on the electrode. Among others, *n* is accumulated as all the additive Ir atoms.

### XAFS Measurements and Analysis

The X‐ray absorption fine structure spectra (Ir *L*3‐edge) were collected at Taiwan Synchrotron Radiation Facility (BSRF). The storage rings of BSRF were operated at 2.5 GeV with a maximum current of 250 mA. Using Si(111) double‐crystal monochromator, the data collection was carried out in transmission mode using an ionization chamber. All spectra were collected in ambient conditions. The acquired EXAFS data were processed according to the standard procedures using the ATHENA module implemented in the IFEFFIT software packages. The *k* ^3^‐weighted EXAFS spectra were obtained by subtracting the post‐edge background from the overall absorption and then normalizing with respect to the edge‐jump step.^[^ ^59^, ^60^ ^]^ Subsequently, *k* ^3^‐weighted *χ*(k) data of Ir L‐edge were Fourier transformed to real (*R*) space using hanging windows (d*k* = 1.0 Å^–1^) to separate the EXAFS contributions from different coordination shells. The least‐squares curve parameter fitting was performed using the ARTEMIS module of IFEFFIT software packages to obtain the quantitative structural parameters around central atoms.^[^ ^61^ ^]^

### Theoretical Calculation

Spin‐polarized DFT calculations were performed with the projected augmented wave method, as implemented in the Vienna Ab‐initio Simulation Package.^[^ ^62^, ^63^ ^]^ The exchange‐correction function was treated by the generalized gradient approximation (GGA) of Perdew−Burke−Ernzerhof functional, and the wave functions were expanded on a plane wave basis with an energy cutoff of 500 eV. The effective *U–J* values of 3.9, 6.2, and 0 eV were introduced to account for the strong on‐site Coulomb repulsion of Mn, Ni, and Ir (no *U* correction) atoms, respectively.^[^ ^64^, ^65^ ^]^ The gamma‐centered scheme for K‐points grid sampling was applied for all the calculations. For all the calculations, the convergence criteria for the electronic and ionic relaxations are 10^−5^ eV and 0.02 eV Å^−1^, respectively.

## Conflict of Interest

The authors declare no conflict of interest.

## Supporting information

Supporting Information

## Data Availability

The data that support the findings of this study are available from the corresponding author upon reasonable request.
