# Supplementary material for: Large‐Scale Synthesis of Spinel NixMn3‐xO4 Solid Solution Immobilized with Iridium Single Atoms for Efficient Alkaline Seawater Electrolysis
Source: Adv Sci (Weinh). 2022 Mar 27;9(16):2200529. doi: 10.1002/advs.202200529 (PMC9165520; doi:10.1002/advs.202200529)
Supplement: Supplementary file 1 — Supporting Information [file ADVS-9-2200529-s001.pdf]

ADVANCED SCIENCE
Open Access

Supporting Information

for *Adv. Sci.*, DOI 10.1002/advs.202200529

Large-Scale Synthesis of Spinel $Ni_xMn_{3-x}O_4$ Solid Solution Immobilized with Iridium Single Atoms for Efficient Alkaline Seawater Electrolysis

Ning Wen, Yuguo Xia, Haihua Wang, Dongpeng Zhang, Haimei Wang, Xiang Wang, Xiuling Jiao and Dairong Chen**

Supporting Information

Large-Scale Synthesis of Spinel $Ni_xMn_{3-x}O_4$ Solid Solution Immobilized with Iridium Single Atoms for Efficient Alkaline Seawater Electrolysis

Ning Wen,[a] Yuguo Xia,[a] Haihua Wang,[a] Dongpeng Zhang,[b] Haimei Wang,[a] Xiang Wang,[a] Xiuling Jiao,[a] and Dairong Chen*[a]*

Computational models:

The theoretical models for DFT calculations are constructed as the following three steps:

Step1: The primary cells of $NiMn_2O_4$ and Ni_2MnO_4 are optimized, and we find $NiMn_2O_4$ with an antiferromagnetic configuration possess lower total energy.

Step2: According to the experimental LSV curves, we find that both the $Ni_{1.6}Mn_{1.4}O_4$ and $Ir_1/Ni_{1.6}Mn_{1.4}O_4$ reveal the best OER activity. Therefore, to elucidate the underlying reasons for the enhanced OER activity, electronic structural changes for $Ni_{1.6}Mn_{1.4}O_4$ and Ir_1/ $Ni_{1.6}Mn_{1.4}O_4$ are mainly concerned. Thus, based on the optimized Ni_2MnO_4 cell, $Mn_8Ni_{16}O_{32}$ (8 unit cells) was constructed. Meanwhile, we find that substituting 3 Ni atoms with Mn atoms to obtain an atom structure of Ni_{13} $Mn_{11}O_{32}$($Ni_{1.625}Mn_{1.375}O_4$) is approaching the experimental crest ($Ni_{1.6}Mn_{1.4}O_4$) of the volcano plot. According to the structural symmetry of cubic spinel, four possible geometry for Ni_{13} $Mn_{11}O_{32}$ were constructed, and the most energetically stable geometry was considered as the optimized cell of Ni_{13} $Mn_{11}O_{32}$(**Figure S5**). Thus, G1 revealed the lowest ground state energy and was employed as optimized Ni_{13} $Mn_{11}O_{32}$.

Step3: According to the experimental HRTEM image and theoretical model predicted by BFDH methods, $(0\bar{1}1)$ crystal plane of $Ni_{1.6}Mn_{1.4}O_4$ dominates. Therefore, $(0\bar{1}1)$ crystal planes were cleaved. Given the effect of truncated atoms on the surface energy, the surface energies of $(0\bar{1}1)$ with different truncated atoms were calculated(**Figure S21**, **Table S3**).

Surface energy calculation

The surface energy is calculated according to the following equation: [1]

$$\gamma = \frac{1}{2A}(E_s^{unrelax} - NE_b) + \frac{1}{A}(E_s^{relax} - E_s^{unrelax}) \qquad (S1)$$

Where A is the surface area, E_s^{relax} and $E_s^{unrelax}$ are separately the total energy of the relaxed and unrelaxed slabs, and E_b is the total energy of the $Ni_{1.6}Mn_{1.4}O_4$ unit cell.

OER mechanism calculation.
The OER process proceeding through a four-electron pathway in alkaline media is summarized as the following elementary steps: [2]

$$*+OH^- \rightarrow *OH+e^- \quad (S2)$$

$$*OH+OH^- \rightarrow *O+H_2O+e^- \quad (S3)$$

$$*O+OH^- \rightarrow *OOH+e^- \quad (S4)$$

$$*OOH+OH^- \rightarrow *+O_2+H_2O+e^- \quad (S5)$$

Where the asterisk (*) stands for the active site on the catalyst, while *O, *OH, and *OOH represent the intermediates during the OER evolution.

For each step, the reaction Gibbs free energy (ΔG) is defined as the difference between free energies of the initial and final states and is given by the expression,

$$\Delta G=\Delta E+\Delta ZPE-T\Delta S-eU \quad (S6)$$

Where the ΔE is the reaction energy of reaction and product molecules absorbed on catalyst surface obtained from DFT calculations, ΔZPE is the difference of zero-point energy, T equals to 298.15 K, ΔS is the entropy change, and U is the potential applied at the electrode. Of note, total energies of clean $Ni_{1.6}Mn_{1.4}O_4$-$(0\bar{1}1)$and Ir_1-$Ni_{1.6}Mn_{1.4}O_4$-$(0\bar{1}1)$, as well as the energies of the most stable O*, OH*, OOH* species absorption geometries, are provided in **Table S4**. The energies of $H_2O(l)$ and H_2 (g) are referenced to Nørskov's work,[1] and frequencies of adsorbed species, which are calculated for the zero-point energy (ZPE) corrections, are supplemented in **Table S5**.

The overpotential (η_{OER}) for OER can be obtained by eq S7.[2]

$$\eta_{OER}=\frac{\max\{\Delta G_1,\Delta G_2,\Delta G_3,\Delta G_4\}}{e}-1.23V \quad (S7)$$

Where ΔG_1, ΔG_2, ΔG_3, and ΔG_4 are the free energies of reactions (S2-S5), respectively.

Supplementary figures

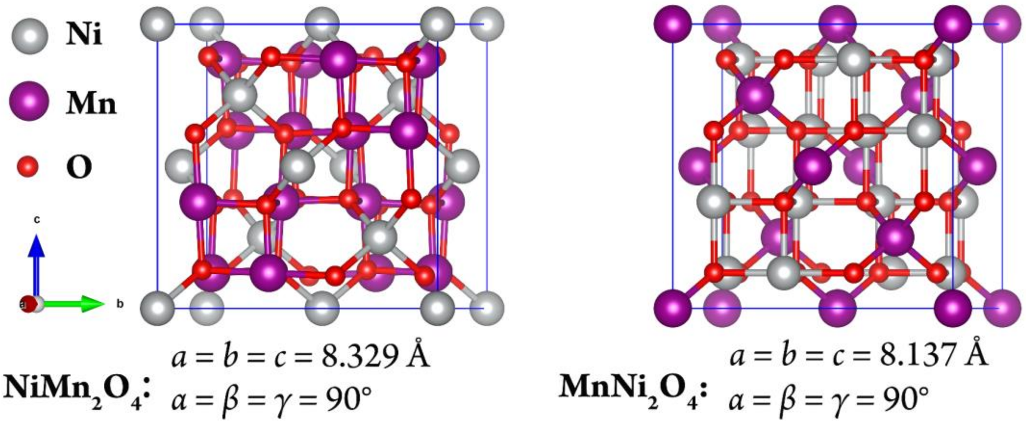

Figure S1. Optimized structures of $NiMn_2O_4$ and $MnNi_2O_4$.

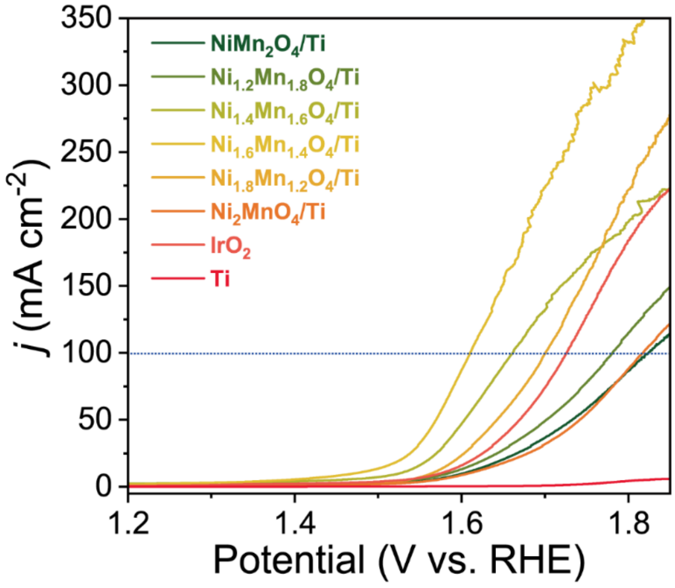

Figure S2. Polarization curves of $Ni_xMn_{3-x}O_4$ solid solutions in 1 M KOH.

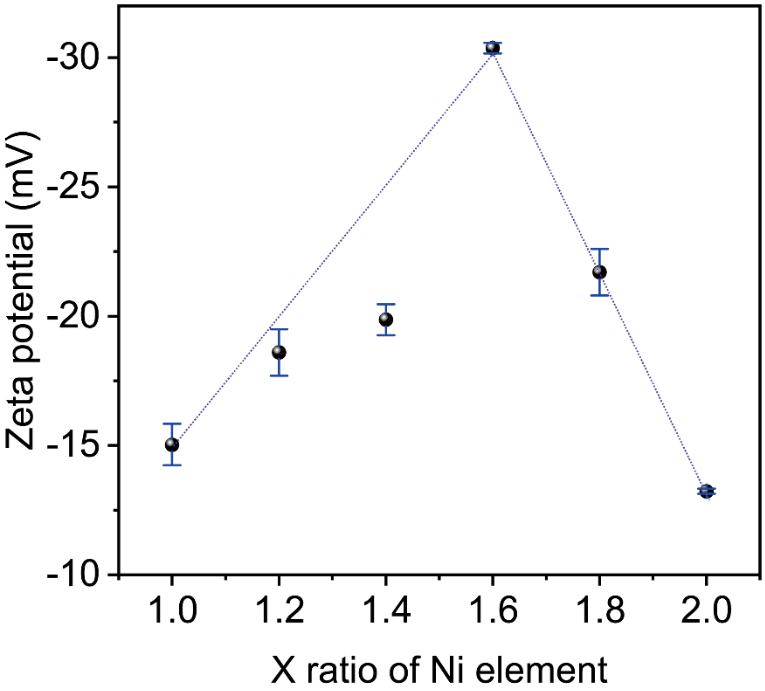

Figure S3. The surface charge of $Ni_xMn_{3-x}O_4$.

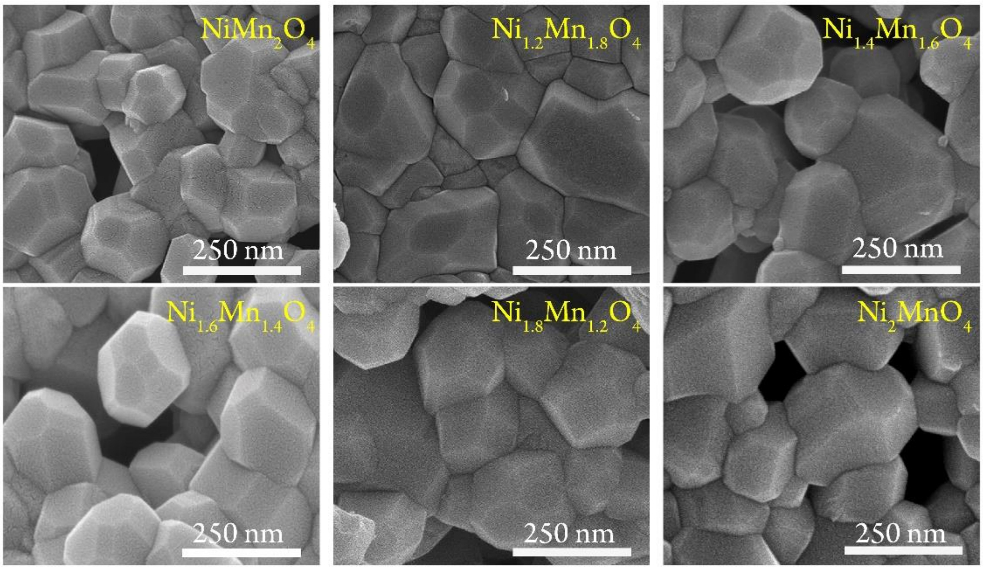

Figure S4. The SEM images of $Ni_xMn_{3-x}O_4$.

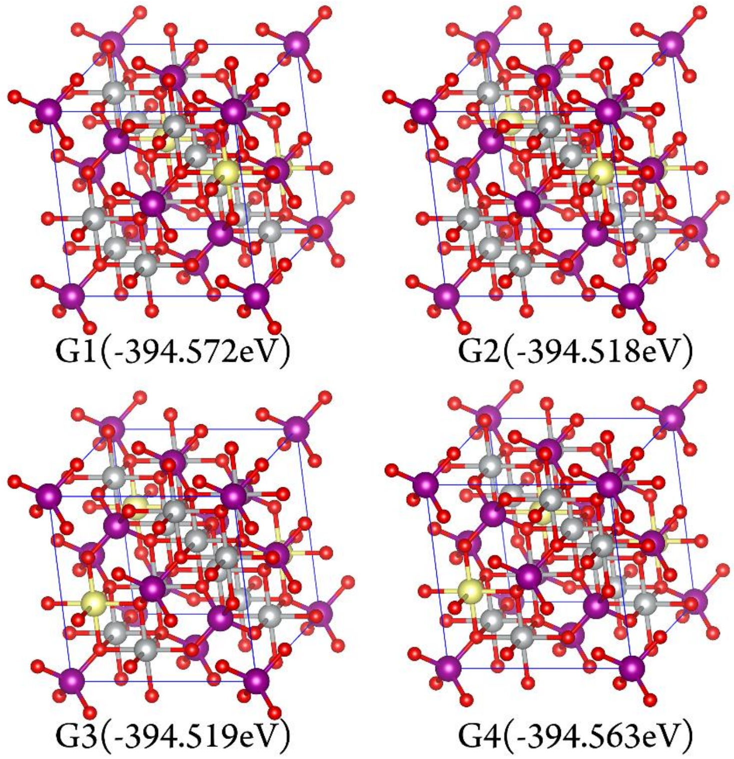

Figure S5. The optimized cell structure for $Ni_{1.625}Mn_{1.375}O_4$.

The G1 geometry revealed the lowest ground state energy and was employed as the theoretical model to simulate $Ni_{1.6}Mn_{1.4}O_4$.

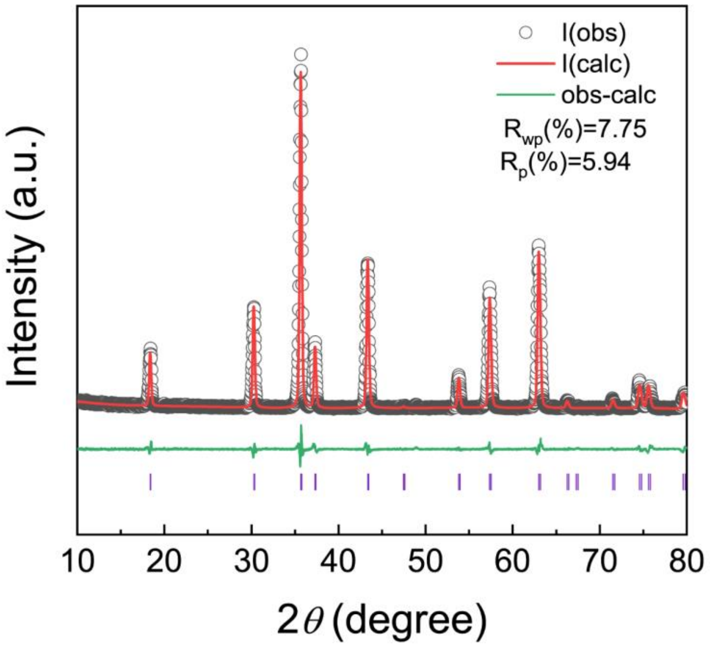

Figure S6. Rietveld analysis for $Ni_{1.6}Mn_{1.4}O_4$.

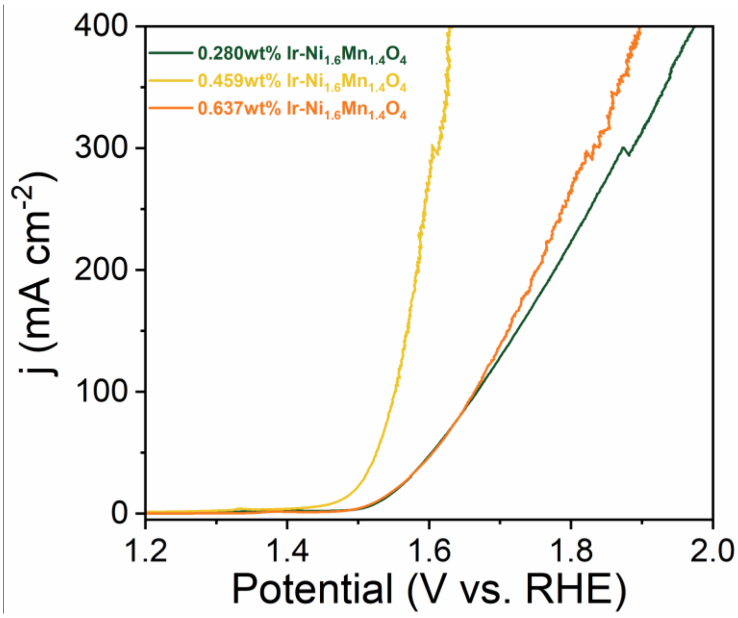

Figure S7. OER polarization curves for Ir-$Ni_{1.6}Mn_{1.4}O_4$ with different loading amounts of Ir.

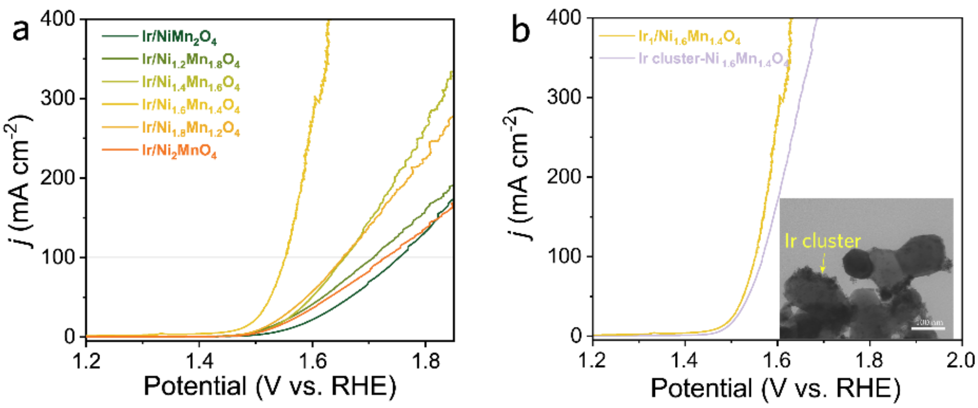

Figure S8. LSV curves of (a) Ir/$Ni_xMn_{3-x}O_4$ and (b) Ir cluster/$Ni_{1.6}Mn_{1.4}O_4$. The inset of (b) was the corresponding TEM image.

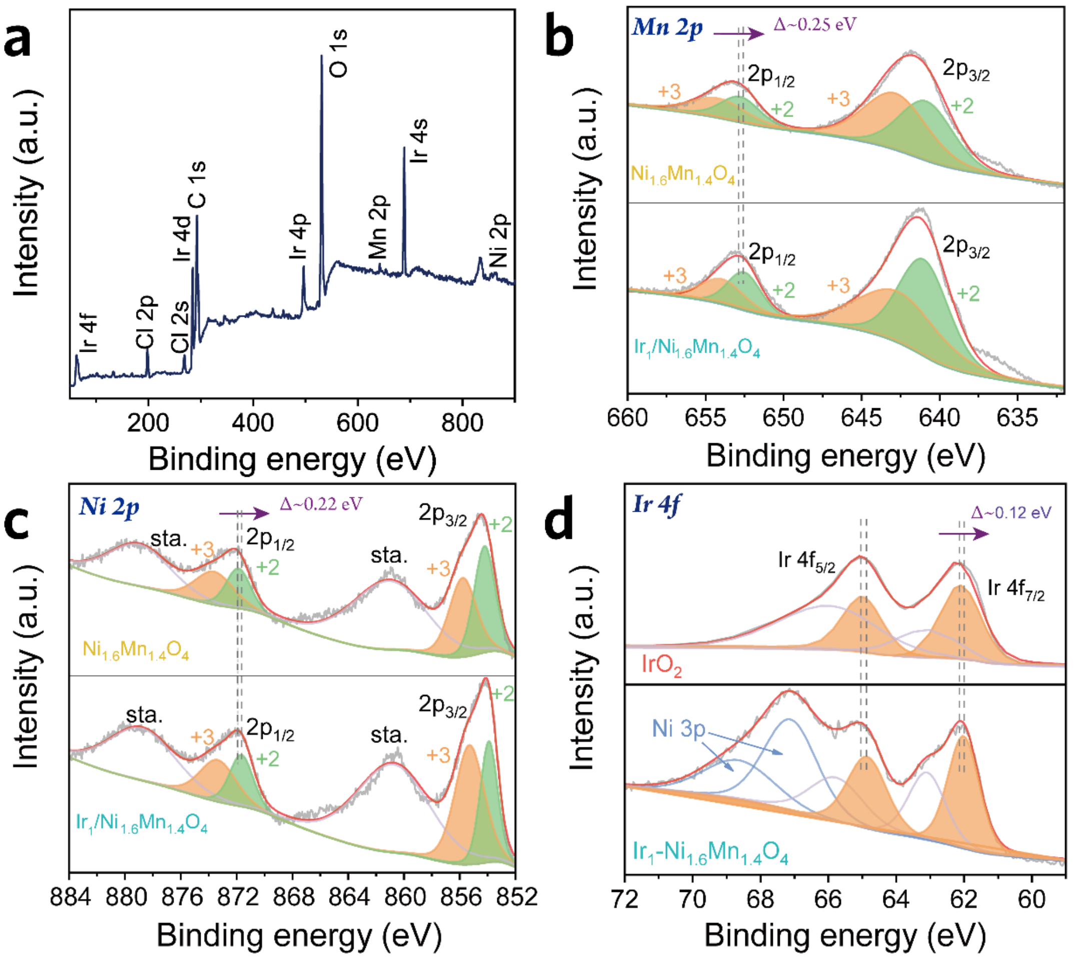

Figure S9. (a) XPS survey spectrum. (b-d) core-level XPS spectra of Mn 2p, Ni 2p, and Ir 4f for $Ir_1/Ni_{1.6}Mn_{1.4}O_4$.

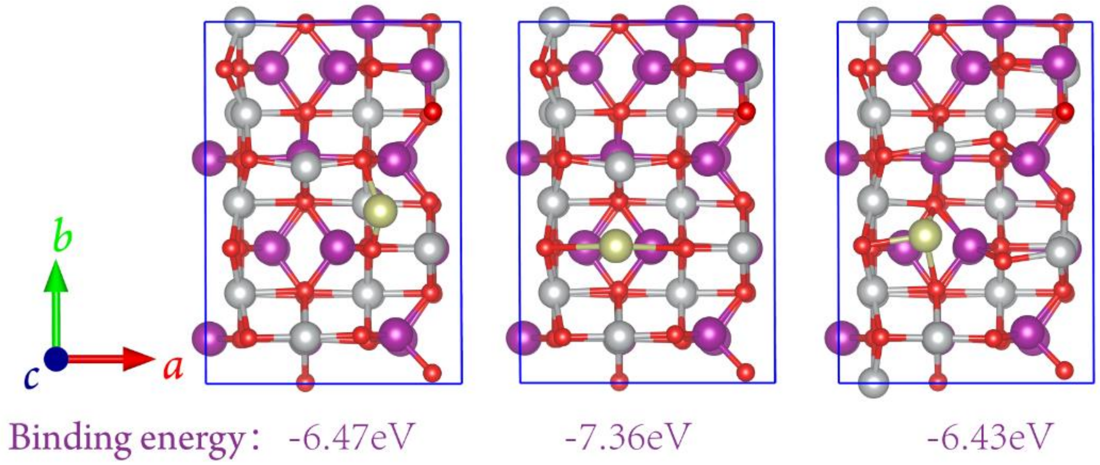

Figure S10. Possible geometries of Ir absorbing on $Ni_{1.6}Mn_{1.4}O_4$-$(0\bar{1}1)$ and corresponding binding energies.

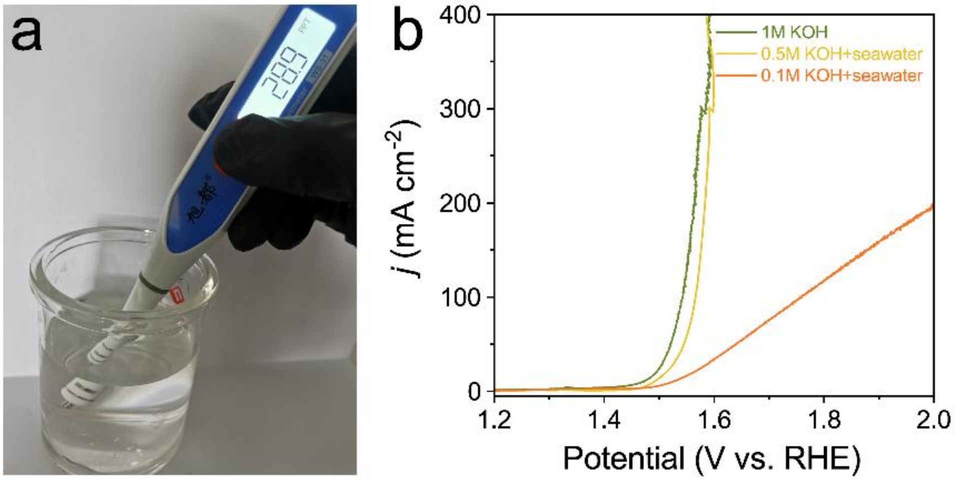

Figure S11. (a) The salinity test for the natural seawater and (b) OER polarization curves for Ir-$Ni_{1.6}Mn_{1.4}O_4$ in alkaline seawater with different alkalinity. The salinity of the seawater sampled from Qingdao, China, is 28.9 PPT, corresponding to ca. 0.5 M NaCl solution.

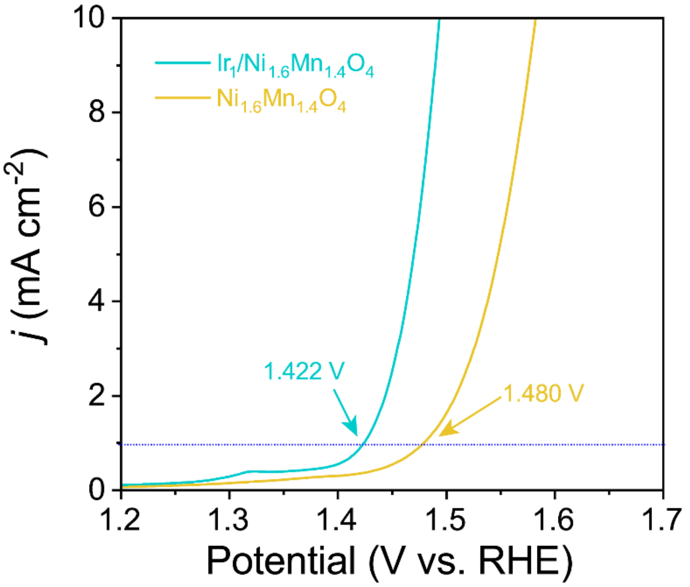

Figure S12. The onset potentials determination for $Ni_{1.6}Mn_{1.4}O_4$ and $Ir_1/Ni_{1.6}Mn_{1.4}O_4$ at the current density of 1 mA cm^{-2}.

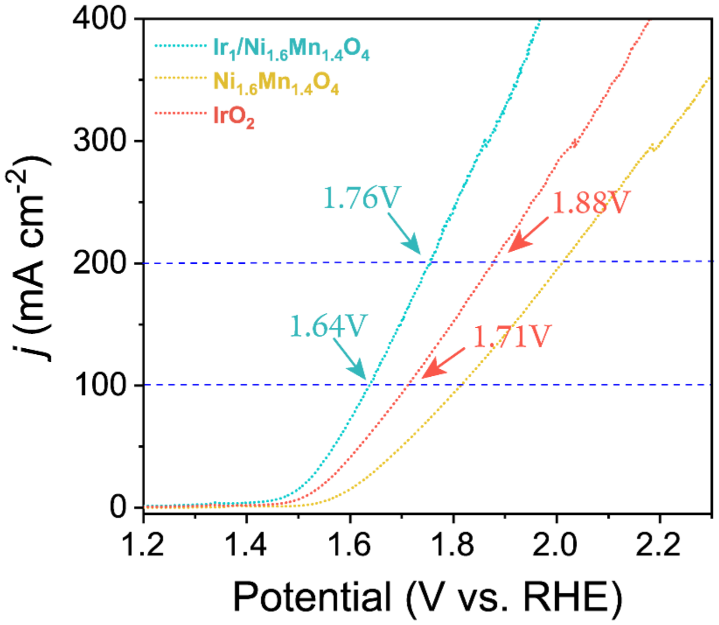

Figure S13. The LSV polarization curves for $Ni_{1.6}Mn_{1.4}O_4$ and $Ir_1/Ni_{1.6}Mn_{1.4}O_4$ without *i*R compensation.

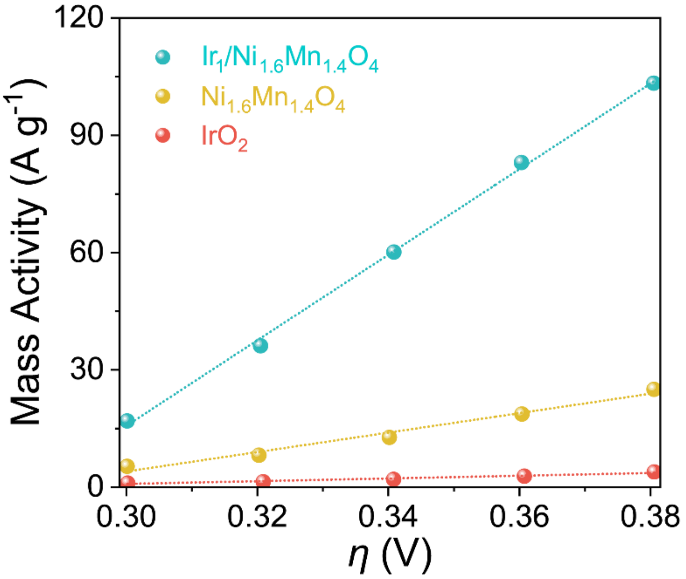

Figure S14. The mass activity of $Ir_1/Ni_{1.6}Mn_{1.4}O_4$ compared to pristine $Ni_{1.6}Mn_{1.4}O_4$ and commercial IrO_2.

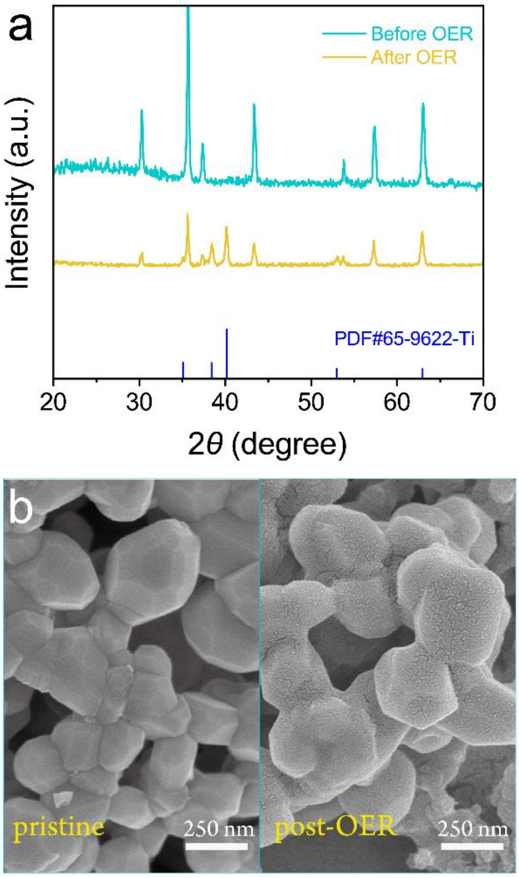

Figure S15. XRD patterns and SEM images of $Ir_1/Ni_{1.6}Mn_{1.4}O_4$ after 60 h OER test compared to the pristine sample.

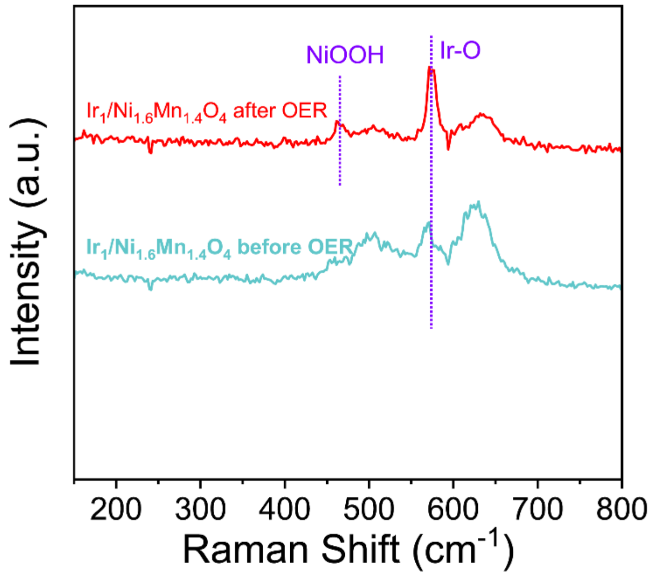

Figure S16. Raman spectra of pristine and recycled $Ir_1/Ni_{1.6}Mn_{1.4}O_4$.

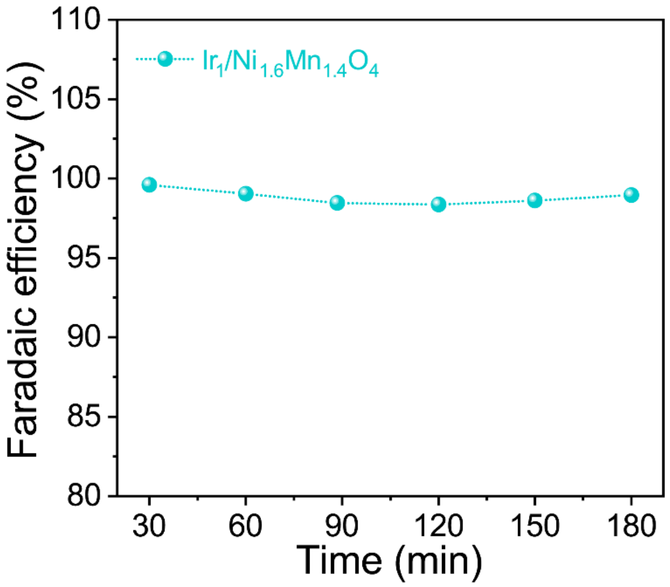

Figure S17. Faradaic efficiency of $Ir_1/Ni_{1.6}Mn_{1.4}O_4$ during OER measurement.

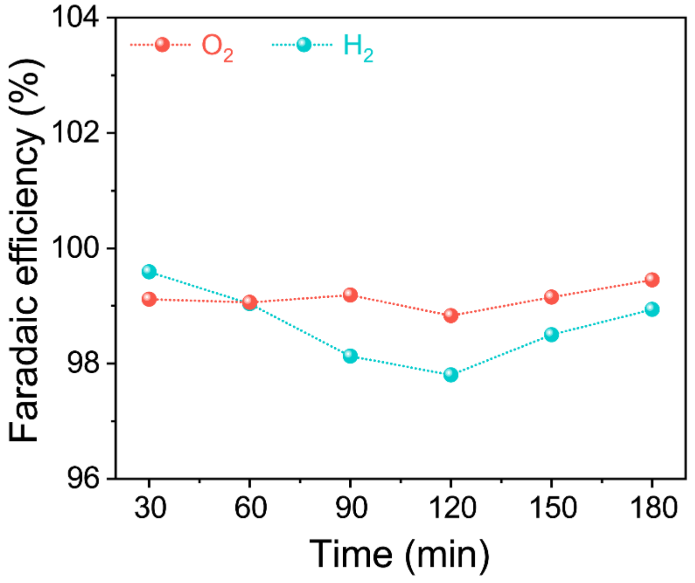

Figure S18. Faradaic efficiency of $Ir_1/Ni_{1.6}Mn_{1.4}O_4$ ‖ Pt/C catalytic couple during OER measurement.

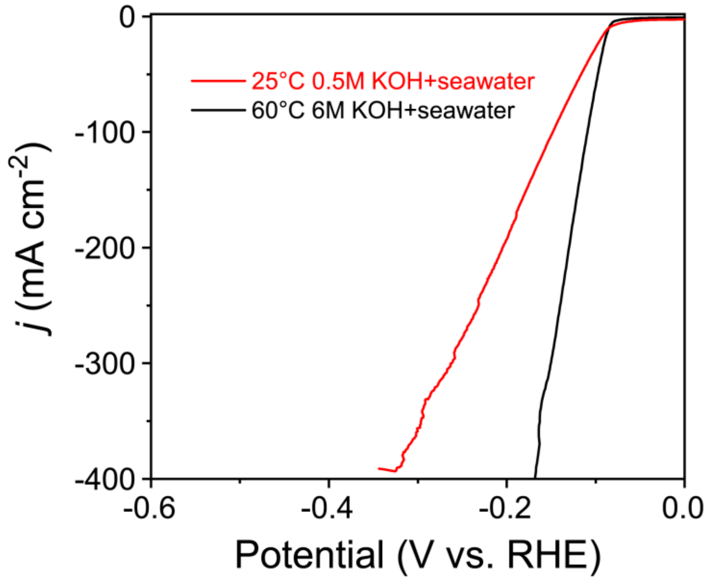

Figure S19. The LSV curves of Pt/C in 0.5 M KOH +seawater at 25°C and 6 M KOH+ seawater at 60 °C with *i*R correction.

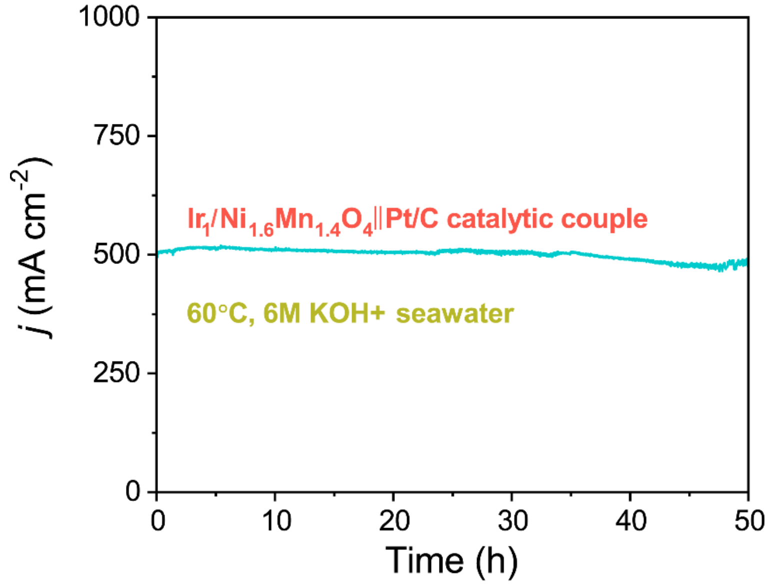

Figure S20. Long-term stability of $Ir_1/Ni_{1.6}Mn_{1.4}O_4$ ‖ Pt/C catalytic couple in 6 M KOH+seawater at 60˚C

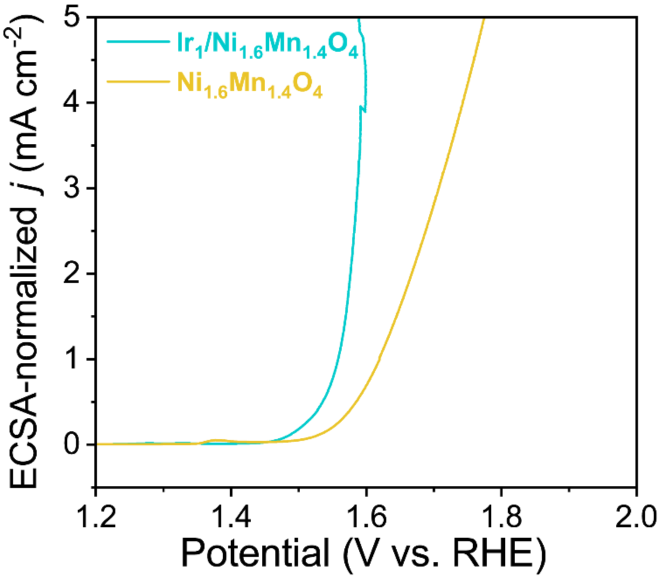

Figure S21. ECSA-normalized OER polarization curves.

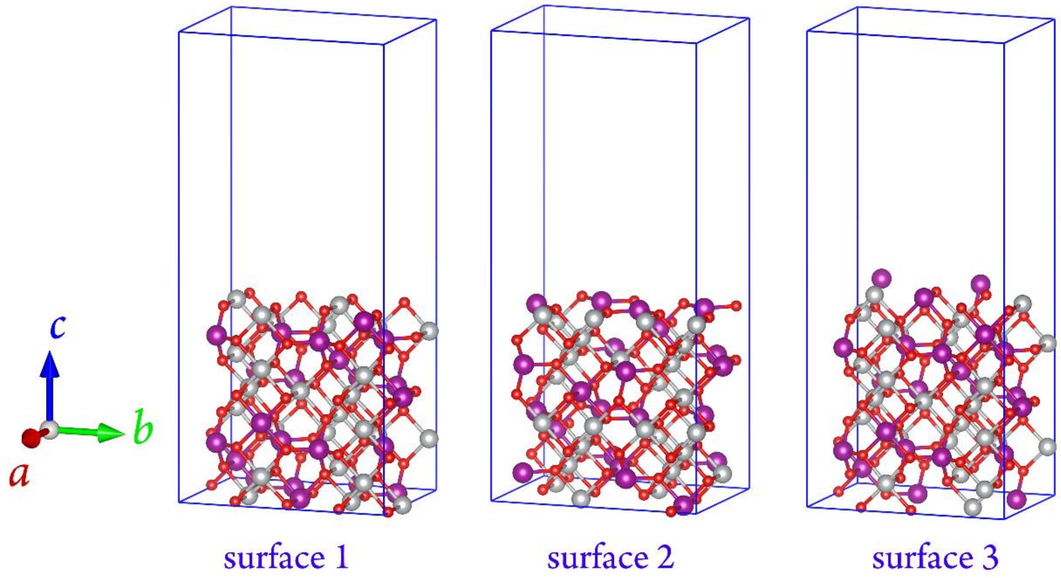

Figure S22. Geometries of $Ni_{1.6}Mn_{1.4}O_4$-$(0\bar{1}1)$ with different truncated atoms.

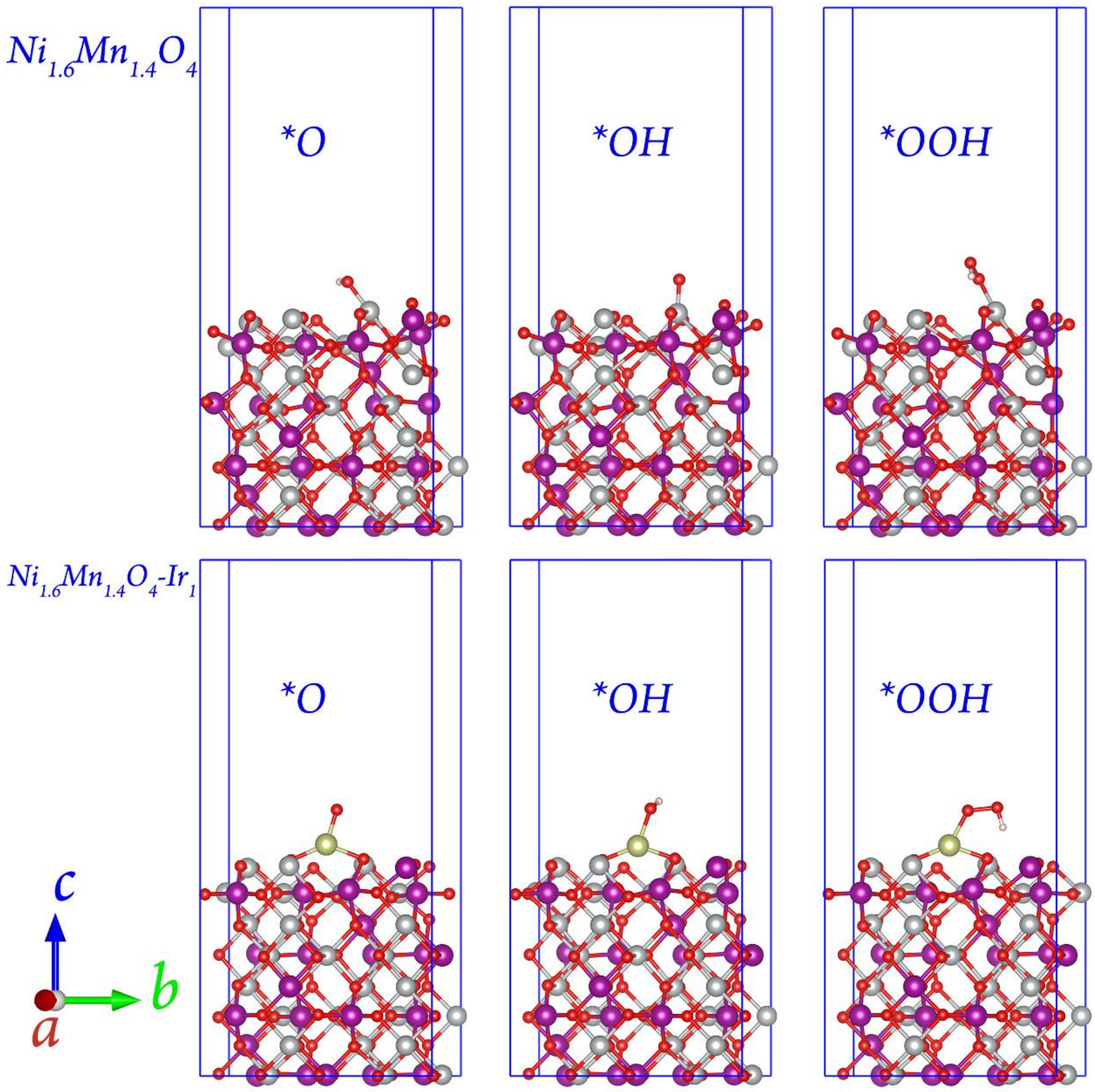

Figure S23. The optimized geometries of *O, *OH, and *OOH intermediates absorbing on $Ni_{1.6}Mn_{1.4}O_4$-$(0\bar{1}1)$ and Ir_1-$Ni_{1.6}Mn_{1.4}O_4$-$(0\bar{1}1)$.

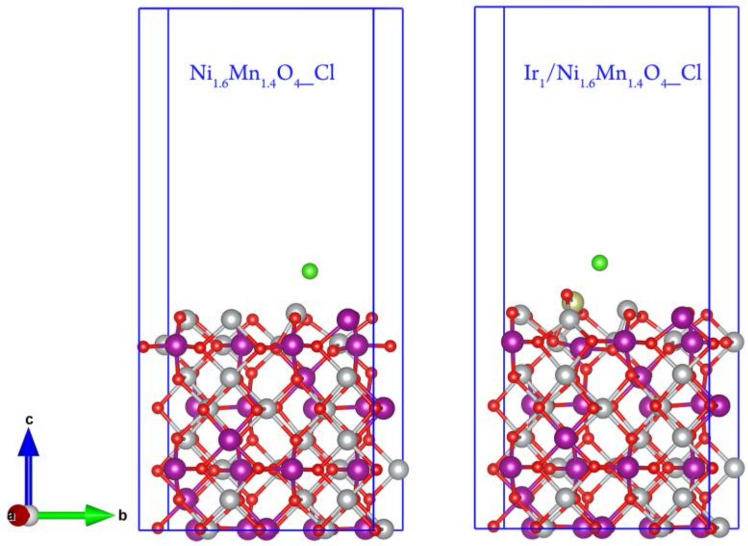

Figure S24. The optimized geometries of Cl^- absorbing on $Ni_{1.6}Mn_{1.4}O_4$ and $Ir_1/Ni_{1.6}Mn_{1.4}O_4$

Supplementary Tables

Table S1. Elemental analysis for $Ir_1/Ni_{1.6}Mn_{1.4}O_4$ by ICP-MS.

**Elements**	Ir	Ni	Mn
**Weight%**	0.459	39.870	31.984

Table S2 . The EXAFS fitting parameters at the Ir K-edge for the $Ir_1/Ni_{1.6}Mn_{1.4}O_4$ sample.

Sample	Shell	*N* [a]	*R* (Å) [b]	σ^2 (Å2·10^{-3}) [c]	ΔE_0 (eV) [d]	*R* factor
Ir	Ir-O	1.9*	2.052-/+0.018	5.79-/+0.57	7.88-/+2.31	0.014

Note: [a] *N*: coordination numbers; [b] *R*: bond distance; [c] σ^2: Debye-Waller factors; [d] ΔE_0: the inner potential correction. *R* factor: goodness of fit.

Table S3. Surface energy calculation for $Ni_{1.6}Mn_{1.4}O_4$-$(0\bar{1}1)$ with different truncated atoms.

Surfaces	E_s^{relax} (eV)	E_b (eV)	$E_s^{unrelax}$ (eV)	Area/ Å^2	Surface energy γ (mJ m^{-2})
Surf1	-746.603	-385.130	-733.827	8.127*11.478	934.4
Surf2	-755.925	-385.130	-751.980	8.127*11.478	892.2
Surf3	-756.562	-385.130	-753.539	8.127*11.478	916.7

Table S4. Total energies of clean $Ni_{1.6}Mn_{1.4}O_4$-(0$\bar{1}$1) and Ir_1-$Ni_{1.6}Mn_{1.4}O_4$-(0$\bar{1}$1) as well as energies of the most stable absorption geometries for *O, *OH, and *OOH intermediates

Surfaces	*E*(*)/eV	*E*(*O)/eV	*E*(*OH)/eV	*E*(*OOH)/eV
$Ni_{1.6}Mn_{1.4}O_4$-(0$\bar{1}$1)	-776.294	-776.859	-783.001	-787.379
Ir_1-$Ni_{1.6}Mn_{1.4}O_4$-(0$\bar{1}$1)	-779.746	-785.343	-789.313	-794.447

Table S5. The ZPE and entropy corrections for *O, *OH, and *OOH intermediates on $Ni_{1.6}Mn_{1.4}O_4$-$(0\bar{1}1)$ and Ir_1-$Ni_{1.6}Mn_{1.4}O_4$-$(0\bar{1}1)$.

Surfaces	*ZPE*(*O) /eV	*ZPE*(*OH)/eV	*ZPE*(*OOH)/eV	*TS*(*O)/eV	*TS*(*OH)/eV	*TS*(*OOH)/eV
$Ni_{1.6}Mn_{1.4}O_4$-$(0\bar{1}1)$	0.076	0.411	0.480	0.024	0.073	0.096
Ir_1-$Ni_{1.6}Mn_{1.4}O_4$-$(0\bar{1}1)$	0.107	0.338	0.524	0.068	0.057	0.175

Table S6. Ground state energy calculated by DFT, ZPE, and *TS* correction for chlorine ion adsorbing on $Ni_{1.6}Mn_{1.4}O_4$-$(0\bar{1}1)$and $Ir_1/Ni_{1.6}Mn_{1.4}O_4$-$(0\bar{1}1)$.

	E_{DFT} (eV)	ZPE (eV)	TS (eV)	△G (eV)
$Ni_{1.6}Mn_{1.4}O_4$_$(0\bar{1}1)$	-773.294	0	0	
$Ir_1/Ni_{1.6}Mn_{1.4}O_4$_$(0\bar{1}1)$	-779.746	0	0	
Cl^-	-4.653	0	0	
$Ni_{1.6}Mn_{1.4}O_4$_Cl	-774.746	0.082	0.124	3.159
$Ir_1/Ni_{1.6}Mn_{1.4}O_4$_Cl	-780.005	0.082	0.121	4.355

References

[1] Q.Li, M.Rellan-Pineiro, N.Almora-Barrios, M.Garcia-Rates, I. N.Remediakis, N. *Nanoscale* **2017,** 9, 13089-13094.

[2] I. C. Man, H. Y.Su, F.Calle-Vallejo, H. A.Calle-Vallejo, J. I.Martínez, N. G.Inoglu, J.Kitchin, T. F.Jaramillo, J. K. Nørskov, J. Rossmeisl, *ChemCatChem* **2011,** 3, 1159-1165.
